# Optimism and the Risk of Developing Dementia

**DOI:** 10.1093/geroni/igaf122.3630

**Published:** 2025-12-31

**Authors:** Säde Stenlund, Hayami Koga, Peter James, Justin Farmer, Colleen McGrath, Francine Grodstein, Laura Kubzansky

**Affiliations:** Harvard T. H. Chan School of Public Health, Boston, Massachusetts, United States; Harvard T.H. Chan School of Public Health, Boston, Massachusetts, United States; University of California, Davis, California, United States; Harvard T. H. Chan School of Public Health, Boston, Massachusetts, United States; Harvard T. H. Chan School of Public Health, Boston, Massachusetts, United States; Rush University Medical Center, Chicago, Illinois, United States; Harvard T. H. Chan School of Public Health, Boston, Massachusetts, United States

## Abstract

Previous studies suggest that higher optimism is associated with better cognitive function and slower cognitive decline in aging. Using data from the Health and Retirement Study, a nationally representative sample of older U.S. adults, we examined whether optimism was associated with lower risk of developing dementia in different population groups and if it was maintained after accounting for initial health status and other potential confounders as well as in critical sensitivity analyses. Optimism was measured in 9,071 cognitively healthy individuals within 2 years before each person’s analytic baseline using the validated Life Orientation Test-Revised and dementia onset was identified by an algorithm developed to perform well across major racial and ethnic groups over eight waves of data collection in 2006–2020. Using Cox proportional hazards models, we observed that a 1-standard deviation increase in optimism was associated with a lower hazard of developing dementia (hazard ratio = 0.82, 95% confidence interval 0.79–0.85) over follow-up ranging up to 14 years. Associations did not substantially change when adjusting for age, sex, race and ethnicity, education, depression, major health conditions, health behaviors and depression, or when we removed the first two years of follow-up to address reverse causation or excluded individuals with poorest mental health. When stratifying by race and ethnicity, we observed similar associations in the Non-Hispanic White and Black sub-populations. Findings suggest optimism may be a novel target for interventions seeking to lower the risk of dementia.

